# 29 Mammalian Genomes Reveal Novel Exaptations of Mobile Elements for Likely Regulatory Functions in the Human Genome

**DOI:** 10.1371/journal.pone.0043128

**Published:** 2012-08-27

**Authors:** Craig B. Lowe, David Haussler

**Affiliations:** 1 Center for Biomolecular Science and Engineering, University of California Santa Cruz, Santa Cruz, California, United States of America; 2 Howard Hughes Medical Institute, University of California Santa Cruz, Santa Cruz, California, United States of America; Academia Sinica, Taiwan

## Abstract

Recent research supports the view that changes in gene regulation, as opposed to changes in the genes themselves, play a significant role in morphological evolution. Gene regulation is largely dependent on transcription factor binding sites. Researchers are now able to use the available 29 mammalian genomes to measure selective constraint at the level of binding sites. This detailed map of constraint suggests that mammalian genomes co-opt fragments of mobile elements to act as gene regulatory sequence on a large scale. In the human genome we detect over 280,000 putative regulatory elements, totaling approximately 7 Mb of sequence, that originated as mobile element insertions. These putative regulatory regions are conserved non-exonic elements (CNEEs), which show considerable cross-species constraint and signatures of continued negative selection in humans, yet do not appear in a known mature transcript. These putative regulatory elements were co-opted from SINE, LINE, LTR and DNA transposon insertions. We demonstrate that at least 11%, and an estimated 20%, of gene regulatory sequence in the human genome showing cross-species conservation was co-opted from mobile elements. The location in the genome of CNEEs co-opted from mobile elements closely resembles that of CNEEs in general, except in the centers of the largest gene deserts where recognizable co-option events are relatively rare. We find that regions of certain mobile element insertions are more likely to be held under purifying selection than others. In particular, we show 6 examples where paralogous instances of an often co-opted mobile element region define a sequence motif that closely matches a transcription factor’s binding profile.

## Introduction

By comparing the genomes of 29 eutherian mammals, researchers estimate that at least 5.5% of the human genome has been evolving under purifying selection and is therefore functional [1], which is similar to previous estimates [2], [3]. Protein-coding exons comprise ∼20% of the bases under selection. Untranslated regions and non-coding RNAs explain an additional ∼7% of bases under selection. This leaves 73% of the bases evolving under constraint in the human genome not appearing in a known mature transcript [1]. These conserved non-exonic elements (CNEEs) are thought to be largely gene regulatory regions that control the spacial and temporal expression of genes during development [4]–[7], reviewed in [8].

The importance of changes in gene regulatory regions was hypothesized by King and Wilson in 1975 to address the paradox of how humans and chimps could be so different in anatomy and physiology, yet have few differences in the proteins encoded by their genomes [9]. This hypothesis has gained support as a number of differences between closely related species have been shown to be the result of gene regulatory regions being gained, lost, or modified. This includes pigmentation changes in flies, fish, and dogs [10]–[12], bristle patterns in flies [13], and skeletal differences between fish populations [14], [15].

Researchers have discovered that not only does the origin, modification, and deletion of gene regulatory regions have a significant role in morphological changes between closely related species or within a species, but the amount of putative gene regulatory sequence correlates with the perceived complexity of an organism. The amount of conserved non-exonic sequence increases significantly from yeast, to worms, to flies, and to mammals [16]. Despite the importance of changes in gene regulatory sequence and its accumulation as organism complexity increases, we have only recently begun to understand the mechanisms by which new regulatory regions are created.

When Barbara McClintock first discovered transposable elements, she chose to term them “controlling elements” [17], after their ability to influence the expression of nearby genes instead of “transposable elements” or “mobile elements,” which are commonly used today to reference their ability to move locations in the genome. Decades later, in the pre-genomic era, McClintock’s vision of mobile elements controlling the expression of nearby genes was revitalized by researchers who noticed many mobile elements had been co-opted to serve gene regulatory functions for the host [11], [19], a process that fits under the term “exaptation” [20]. There are currently examples of transposon insertions acting as developmental enhancers [21]–[24], which can create new expression patterns for genes [25] or increase the amount of mRNA produced by a gene already expressed in a tissue [26], reviewed in [27], [28]. Some mobile element families have even been shown to play a significant role in modifying the regulatory networks of key transcription factors [29]–[31], or even helping to create new cell types [32].

With the availability of the first mammalian genomes researchers had the opportunity to conduct genome-wide surveys of mobile elements being placed under selection to act as putative gene regulatory elements. An early survey of the human genome noted that 2.5% of cis-regulatory elements known at the time were at least partially composed of mobile element insertions [33]. In later studies the percentage of regulatory regions attributable to mobile elements has been estimated to be as high as 16% [34] and 10% of transcription factor binding sites may have been deposited by mobile elements [35]. There are now 29 mammalian genomes available, more than four times as many as when the previous studies were published. This influx of data allows for a higher resolution analysis of mobile elements contributing to gene regulatory innovations on the human lineage.

## Results

We used a set of conserved, and by extension functional, elements in the human genome that were defined using a phylogenetic hidden Markov model [16] on a multi-species alignment of 29 mammalian genomes [1]. This method identifies regions of the genome with strong cross-species conservation based on a depletion in substitutions. Regions with strong cross-species conservation show evidence of being under strong negative selection and are not explained by low mutation rates [36]. While some regions of the genome have faster or slower mutation rates in extant populations, the locations of these regions are not consistent throughout mammalian lineages [37], [38] and therefore have a minimal effect when looking for regions of the genome exhibiting cross-species conservation. Using cross-species conservation to elucidate functional regions will miss functional elements that are not under strong selection, are lineage specific, or undergo rapid turnover [39], [40]. For these regions the total amount of bases under selection may be two to three times what is seen by cross-species conservation [41].

To create a subset of putative regulatory regions we removed all conserved elements that overlap protein-coding exons, 3′ untranslated regions (UTRs), 5′ UTRs, or exons from non-coding RNA genes that have currently been identified (see Methods). The resulting set of approximately 2.6 million conserved non-exonic elements (CNEEs) totals 75 Mbp, which is 2.6% of the human genome. These CNEEs are under selection, but do not appear in mature transcripts. A previous study demonstrated that 50% of 437 CNEEs tested at a single time point in development acted as tissue specific enhancer elements [42]. CNEEs also act as repressors [43], insulators [44], [45], matrix attachment regions [46], and regulators of splicing [47].

To understand which of these putative regulatory elements in the human genome are the result of mobile element insertions we examined the overlap of our CNEEs with mobile element annotations generated by running RepeatMasker on the human genome (see Methods). We did not keep all CNEEs that overlapped a mobile element insertion, but only those which had a majority of their bases annotated as originating in a SINE, LINE, LTR, or DNA transposon insertion. This resulted in a set of 284,857 conserved non-exonic elements, totaling nearly 7 Mb of sequence, that are likely to have been exapted from mobile element insertions.

More than 11% of CNEEs in the extant human genome have been exapted from mobile element insertions. With ∼280,000 exaptations and ∼4.4 million mobile element fragments in the current reference assembly of the human genome, more than 6% of mobile element fragments show signs of selective constraint for a non-exonic function (Table 1). Not only have mobile elements played a significant role in the evolution of gene regulation on the human lineage, but a non-negligible portion of repeat fragments in the reference genome appear to be under selective constraint.

**Table 1:** The exaptation of mobile element classes and superfamilies.

Class	Superfamily	Exapted Elements	Exapted Bases	Genomic Elements	Genomic Bases	Genomic Elements Per Exaptation	Genomic Bases Per Exapted Base
	L1	67,103	1,963,366	937,370	511,375,943	13.9	260.4
	L2	46,532	946,311	462,005	103,894,644	9.9	109.7
	CR1	19,644	58,6282	60,731	10,855,797	3	18.5
	RTE	6,218	156,338	17,696	3,652,083	2.8	23.3
	Dong-R4	797	25,967	550	120,346	0.6	4.6
	RTE-BovB	398	12,401	659	74,688	1.6	6
	L1-like	44	1,715	83	6,788	1.8	3.9
LINE		140,760	3,695,873	1,479,094	629,957,456	10.5	170.4
	MIR	61,335	1,122,485	590,380	84,230,914	9.6	75
	Deu	1,815	70,613	1,266	178,943	0.6	2.5
	Alu	1,624	107,958	1,174,518	306,522,171	723.2	2,839.2
	SINE	1,602	66,502	964	161,994	0.6	2.4
	tRNA	1,026	27,838	1,652	229,877	1.6	8.2
SINE		67,418	1,397,359	1,768,780	391,323,899	26.2	280
	hAT-Charlie	23,994	515,751	251,682	44,862,356	10.4	86.9
	TcMar-Tigger	9,024	264,739	102,787	33,907,139	11.3	128
	hAT-Tip100	2,663	64,363	30,206	6,602,950	11.3	102.5
	TcMar-like	2,380	127,346	3,426	624,957	1.4	4.9
	DNA	1,894	88,980	2,750	339,865	1.4	3.8
	TcMar-Mariner	1,496	39,382	16,229	2,815,735	10.8	71.4
	TcMar-Tc2	1,463	34,606	8,083	1,664,901	5.5	48.1
	hAT-Blackjack	1,360	31,624	19,571	3,415,244	14.3	107.9
	hAT	767	12,459	12,421	1,673,724	16.1	134.3
	TcMar	674	17,320	1,940	319,735	2.8	18.4
	PiggyBac-like	458	18,964	239	44,436	0.5	2.3
	hAT-like	323	6,851	3,027	503,467	9.3	73.4
	PiggyBac	80	3,041	2,115	497,959	26.4	163.7
	MuDR	14	1,302	1,972	686,896	140.8	527.5
	Merlin	1	56	55	17,595	55	314.1
DNA		46,561	1,226,696	456,503	97,959,784	9.8	79.8
	ERVL-MaLR	14,468	289,612	343,284	110,688,741	23.7	382.1
	ERVL	8,441	185,880	157,889	56,087,725	18.7	301.7
	ERV1	2,855	81,186	172,636	83,248,758	60.4	1,025.4
	Gypsy	1,815	38,904	10,760	2,295,297	5.9	58.9
	Gypsy-like	1,323	26,101	7,808	1,454,545	5.9	55.7
	LTR	837	22,332	2,196	472,591	2.6	21.1
	ERVL-like	320	6,700	1,782	413,433	5.5	61.7
	ERV	35	579	580	191,020	16.5	329.9
	ERVK	7	271	10,455	8,790,037	1,493.5	32,435.5
LTR		30083	651,379	707,390	263,530,842	23.5	404.5
Total		284,857	6,988,191	4,411,767	1,382,528,004	15.4	197.8

We show the contribution of mobile elements as a whole, as well as the various classes and superfamilies, to the creation of putative gene regulatory elements on the human lineage. The numbers from the superfamilies do not always add up perfectly to the number for the class. This is because a CNEE is not counted as being exapted from a mobile element unless more than 50% of its bases are annotated as having repeat origins. A CNEE where 45% of the bases are annotated as coming from an L1 insertion and 45% from an L2 insertion will not appear as either an L1 or L2 exaptation, but will be counted as a LINE exaptation. A very small number of bases are also annotated by RepeatMasker as having come from more than one mobile element. Both these situations are rare in our set and the difference in counting never amounts to more than 35 elements.

### CNEEs are Functional in Humans

**Figure 1:**
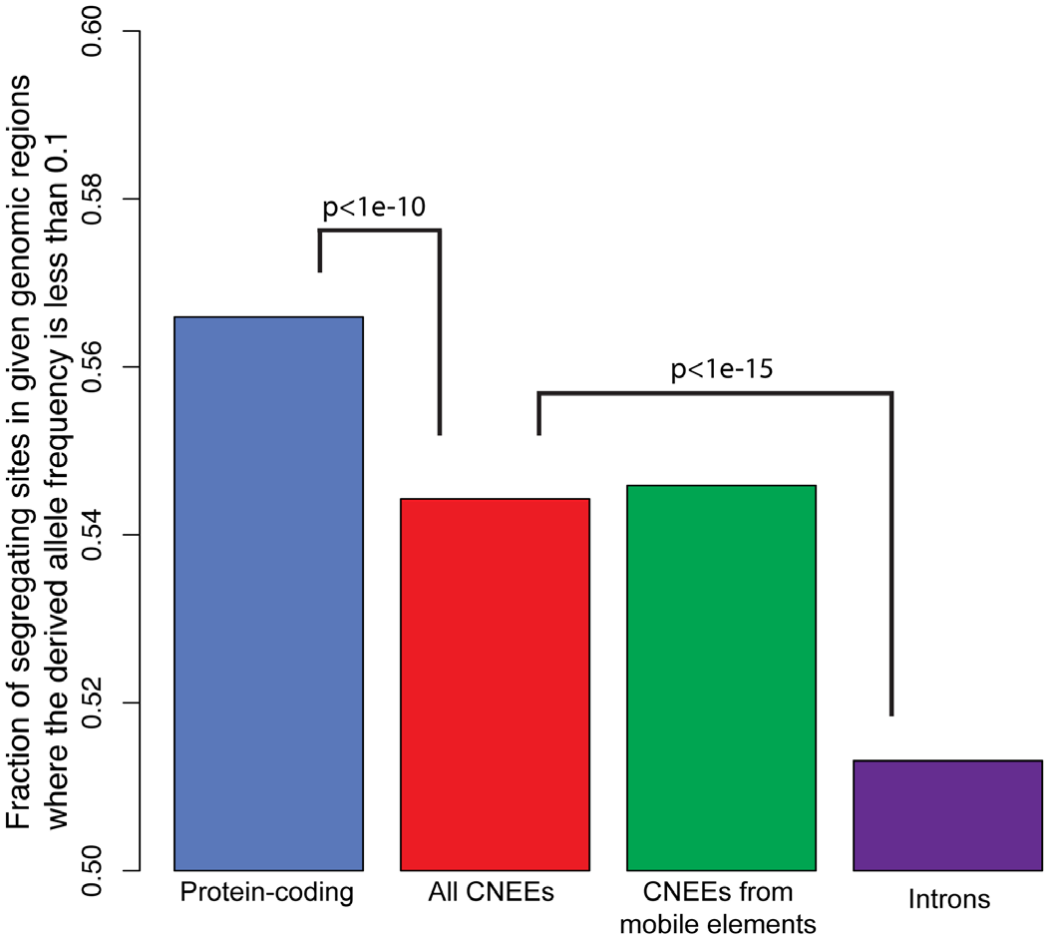
The frequency of rare derived alleles is greater in CNEEs compared to neutral sites. We compared the derived allele frequency spectra for CNEEs as a whole, CNEEs created through the co-option of mobile elements, protein-coding regions, and introns. The spectra representing CNEEs has a lower mean rank of derived allele frequencies, which is indicative of negative selection in humans (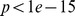, Mann-Whitney U test). However, the selection on these putative regulatory regions does not appear to be as high as that on coding regions (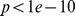, Mann-Whitney U test).

To ensure that the set of CNEEs co-opted by mobile elements is still evolving under constraint in human populations, we examined the derived allele frequency spectrum of single nucleotide polymorphisms currently segregating in the Yoruban population (see Methods). The set of co-opted mobile elements exhibit a characteristic lower mean rank of derived allele frequencies. This shift is indicative of regions under current, or very recent, selective constraint where the majority of mutations are deleterious and rarely progress to high frequencies in the population. While the set of CNEEs as a whole and CNEEs co-opted from mobile elements both show a significant shift relative to intronic regions (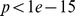, Mann-Whitney U test), which act as a conservative proxy for neutrally evolving sequence, the shift is not as severe as found in protein coding regions (Figure 1). The set of CNEEs co-opted from mobile elements does not show a significant shift relative to the set of CNEEs as a whole (*P* ∼ 0.6) and appears to be under a similar level of constraint in present-day humans.

The set of CNEEs exapted from mobile elements shows enrichments for functional regions identified by biochemical assays. The regions are enriched for transcription factor binding sites (NRSF, 3.1x, 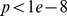; c-Fos, 1.8x, 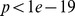; c-Jun, 1.7x, 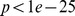; BATF, 1.6x, 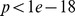; JunD, 1.6x, 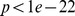; USF1, 1.5x, 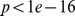; NF-E2, 1.5x, 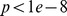; SIX5, 1.5x, 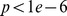), clusters of DNase hypersensitivity sites (1.6x, 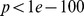), and H3K27 acetylation (1.2x, 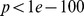) identified by the ENCODE Consortium [48] in human cell lines. Only 25% of the CNEEs from mobile elements were overlapped by a DNase hypersensitivity site; however, some of these regions may only be functional in a different tissue, time point, or environmental condition than that measured by ENCODE, and in some cases there may have been technical difficulties in assaying repetitive regions of the genome [49].

### CNEEs Exapted from Mobile Elements Resemble the Set of all CNEEs

**Figure 2:**
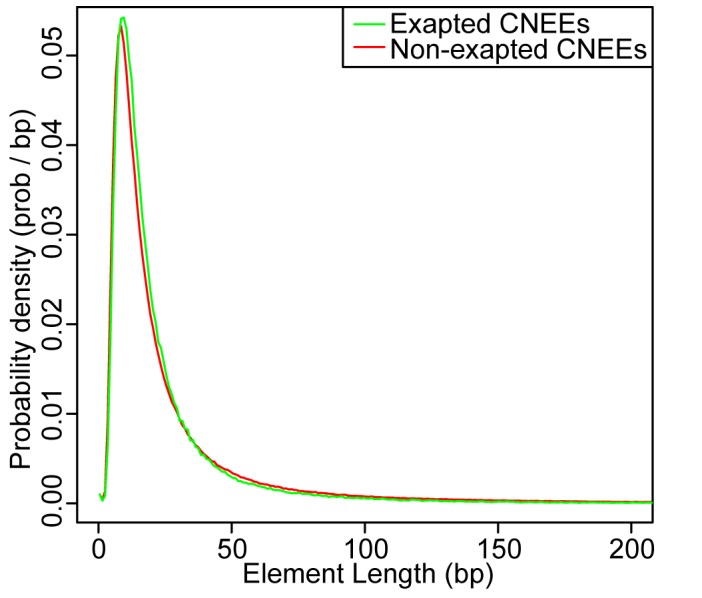
Exapted CNEEs and non-exapted CNEEs have similar length distributions. We compared the entire length of CNEEs where at least half of the bases are annotated as originating in mobile element insertions with those CNEEs not meeting this criteria. The distributions are visually similar, yet have slightly different shapes (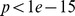, Kolmogorov-Smirnov test). The set of exapted elements has a lower mean length than the non-exapted set, 25 bp and 30 bp respectively, showing a slight depletion of mobile elements depositing very large CNEEs.

The subset of CNEEs exapted from mobile elements has a visually similar distribution of lengths to the set of non-exapted CNEEs (Figure 2). However, the mean of the exapted set is less than that of the non-exapted set, 25 bp and 30 bp respectively, showing a slight bias for the exaptation events to be smaller and the distribution to have a slighty different shape (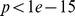, Kolmogorov-Smirnov test). This slight bias towards the exapted elements being smaller may be due to mobile elements being unable to carry very large regulatory modules as many mobile elements are only a few hundred bases in length.

**Figure 3:**
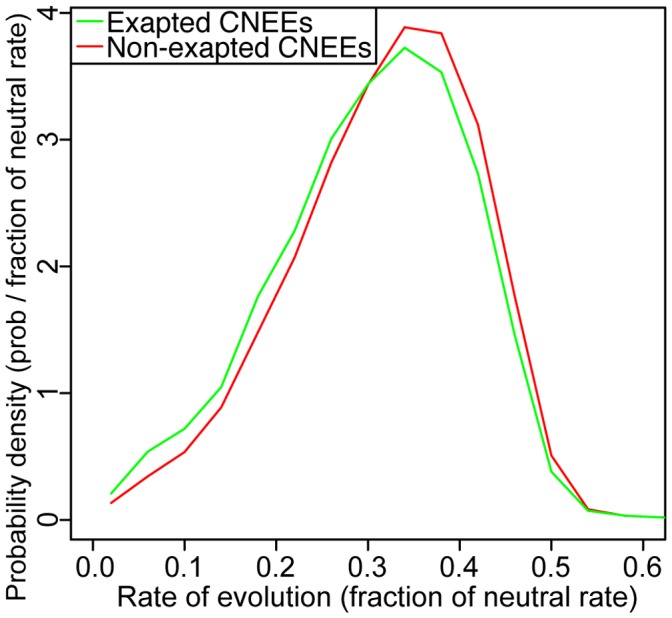
Exapted CNEEs and non-exapted CNEEs have similar distributions of constraint. We calculated the rate of evolution for every CNEE, with respect to the neutral rate, using PhyloFit [82]. The exapted elements evolve with a mean of 0.30 times the neutral rate, while the non-exapted set of CNEEs evolves at 0.32 times the neutral rate. The distributions are visually similarly yet have slightly different shapes (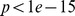, Kolmogorov-Smirnov test) with the exapted elements tending to evolve slightly slower.

The rate of substitution is also visually similar for both sets (Figure 3). However, the exapted elements evolve with a mean of 0.30 times the neutral rate, while the non-exapted set of CNEEs evolves at 0.32 times the neutral rate (see Methods). The distribution of mutational rate has a slightly different shape for the exapted elements (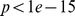, Kolmogorov-Smirnov test). It is possible that this difference is due to a slower rate of evolution, but it is likely due to CNEEs under more severe constraint being closer to the consensus sequence that originally inserted and therefore easier to identify as mobile element insertions.

**Figure 4:**
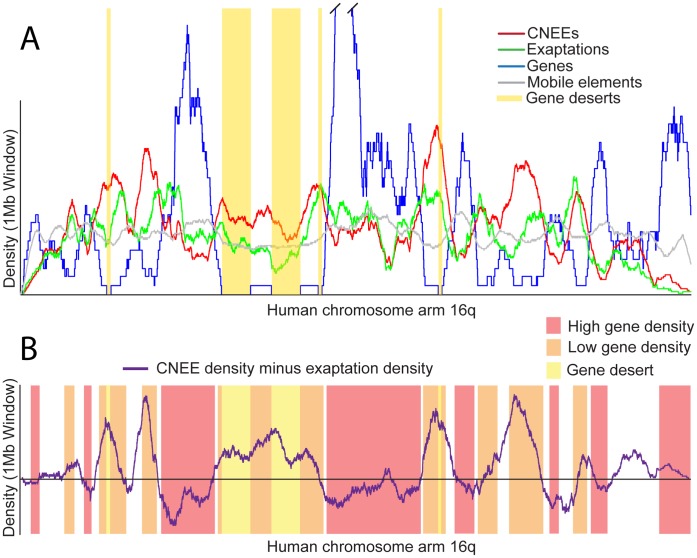
Mobile elements co-opted as conserved non-exonic elements (CNEEs) are rarer than expected in gene deserts. (A) We show the density of genes (blue), all CNEEs (red), just those CNEEs co-opted from mobile elements (green), and mobile elements (gray) windowed over 1 Mb intervals on the q arm of chromosome 16 where there are a number of gene-poor regions. Exaptations are less likely to occur in gene-poor areas when compared to CNEEs in general. (B) The difference between the density of CNEEs and that of exaptations is shown against a schematized backdrop of gene density. CNEEs have a greater normalized density in gene deserts and gene-poor regions of the genome compared to exaptations. In gene deserts, locations in the genome more than 1 Mb from the closest transcription start site, have a depletion of exaptations compared to the number of CNEEs (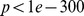, hypergeometric test).

Previous studies used genome-wide enrichment tests to demonstrate that CNEEs exapted from mobile elements cluster near transcription factors and developmental genes [34], [50], which had also been observed for the set of CNEEs as a whole [5], [16], [51]. However, the similarities between the set of all CNEEs and the subset exapted from mobile elements goes beyond clustering near this set of genes. The density plots of the two sets closely correlate with each other (Figure 4). We quantified this similarity by calculating the Pearson product moment correlation coefficient for the changes between the two density functions. The correlation coefficient is 0.55 when comparing those CNEEs originating through exaptation to those originating by other mechanisms. This demonstrates that CNEEs created through the exaptation of mobile elements have similar locations in the genome to those CNEEs originating by other means.

The regions of divergence between the density plots of CNEEs and the subset of CNEEs from mobile element exaptations are rare. The few deviations that do exist consistently happen in the centers of the largest gene deserts (Figure 4). To our knowledge, cis-regulatory elements have only been shown to act over distances of up to 1 Mb from the transcription start site (TSS) of the gene being controlled [52], yet thousands of CNEEs are present in the centers of these large gene deserts, over 1 Mb away from any currently known gene. It is these CNEEs, over 1 Mb away from any known gene, that are rarely found to be exapted from LINEs, SINEs, LTRs, or DNA transposons. Only 1.7% of the CNEEs from exaptation events are more than 1 Mb from the closest TSS, versus 3.1% for non-exapted CNEEs (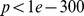, hypergeometric test). This observation holds for both stable gene deserts, which resist rearrangements and have 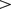
2% of their bases conserved between human and chicken, and variable gene deserts where 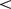
2% of the sequence is conserved [53]. The edges of gene deserts, which harbor large amounts of regulatory material for the developmental genes often found at their borders [54], have an amount of exapted elements in them that reflects the density of CNEEs as a whole, even though the centers of the gene deserts do not.

### Dating Exaptations

We can demonstrate how ancient this process is by explicitly dating each exaptation event. It is possible to date insertions of repetitive elements by analyzing a large multiple alignment of vertebrate species. We assign each insertion to the branch of the human lineage preceding the speciation of the most divergent species that possesses the insertion (see Methods). This method confirms that the exaptation of mobile elements as CNEEs on the human lineage is an ancient process. We detect 133 exaptation events predating the speciation of ray-finned fish from the human lineage, exemplifying that this is a mechanism that has been influential for at least 450 million years [55]. These 133 exaptation events are only identifiable as such because they have been evolving at a slow enough rate and are large enough that they still provide significant alignments to the mobile element consensus that deposited them hundreds of millions of years ago. We also have a poor understanding of the mobile elements that were active at this time since they rarely are active into the present day and their consensus may have changed over time [56], [57]. For these reasons it is likely that many of the CNEEs that were created in our early vertebrate ancestors were deposited by mobile elements, but the exapted area was too small, too quickly evolving, or from a mobile element that was inactivated too long ago for us to realize the origins of these functional elements. Thus, the statistic of over 11% of CNEEs coming from a mobile element insertion is a lower bound of how much mobile elements have contributed to our current repertoire of gene regulation.

**Figure 5:**
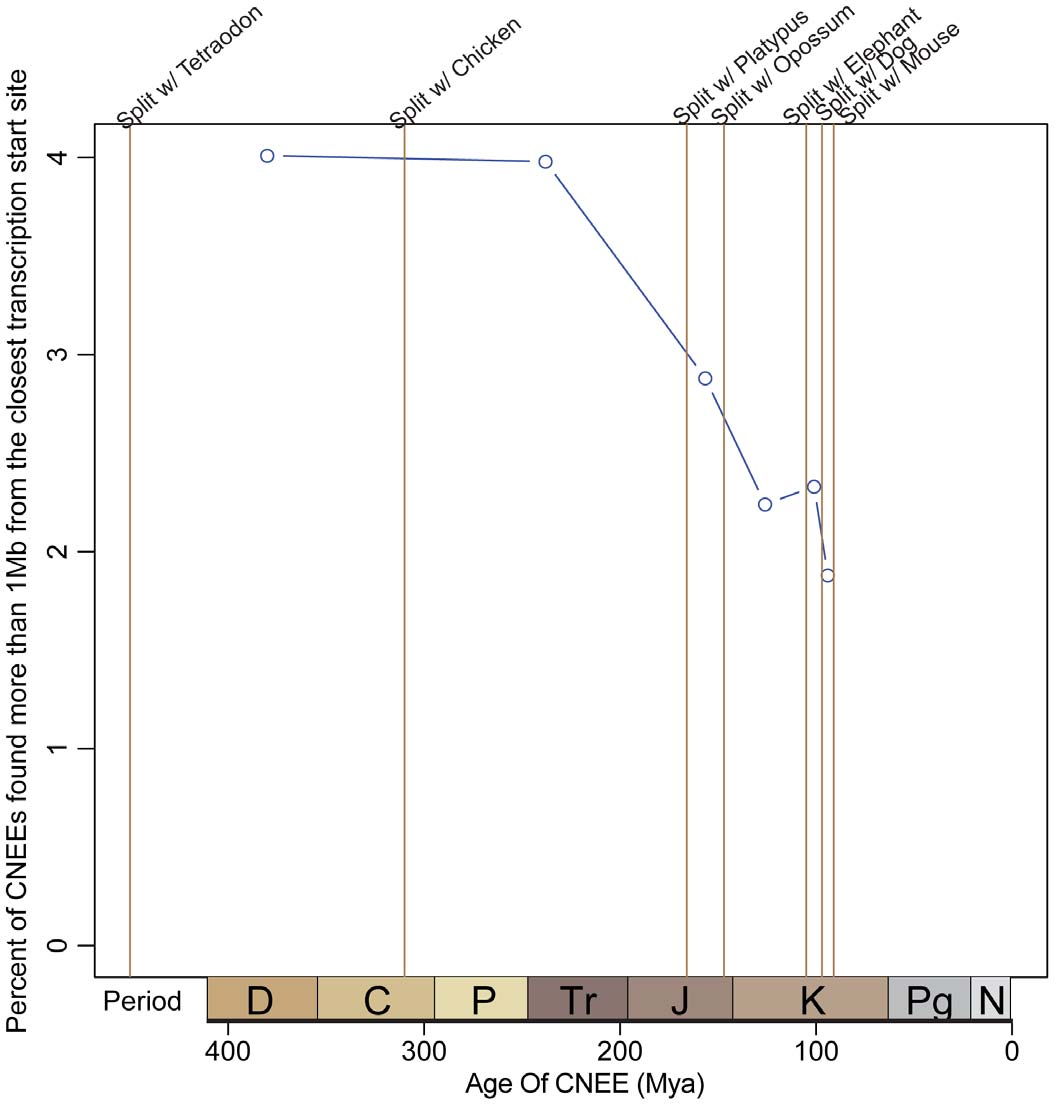
Ancient CNEEs are more likely to be found far from transcription start sites. We infer the branch of origin for all human CNEEs. For the CNEEs originating on each branch we calculate the percentage found more than 1 Mb from the closest transcription start site. Ancient CNEEs are twice as likely to be found far from genes compared to their younger counterparts. Periods: Devonian, Carboniferous, Permian, Triassic, Jurassic, Cretaceous, Paleogene, Neogene.

Using such dating methods, it was shown that the appearance of new CNEEs near different categories of genes has not been uniform during vertebrate evolution [58]. In particular, in early vertebrate evolution, new CNEEs appeared near transcription factors and genes involved in embryonic development twice as frequently as near other types of genes, but this trend ended before the emergence of mammals. Such development-associated genes often flank large gene desserts, so based on this result one might expect an enrichment for ancient CNEEs in large gene desserts, and in particular in the middle of large gene deserts. This is what we find (Figure 5). This tendency for gene deserts to have more ancient CNEEs may explain the observation above that a smaller fraction of CNEEs in these regions come from exaptations of known repetitive elements. This may be due in part to our incomplete knowledge of older mobile element families, which has a disproportionate influence on our statistics in regions that are dominated by ancient CNEEs.

### All Mobile Element Superfamilies Contribute to Regulatory Innovation

Along with analyzing the set of exaptation events as a whole, we can decompose it into subsets based on the class or superfamily of the mobile element that was exapted (Table 1). All 36 superfamilies of LINEs, SINEs, LTRs, and DNA transposons in the human genome have contributed to the increase in putative regulatory material on the human lineage. These repeat superfamilies have been active at various times over the course of vertebrate evolution [56], [57]. The mechanism of the host genome capturing and refining regulatory elements from repeats has not been isolated to one family or one time period in history. This is a process that was happening as far back as we can currently detect mobile element insertions in the human genome.

**Figure 6:**
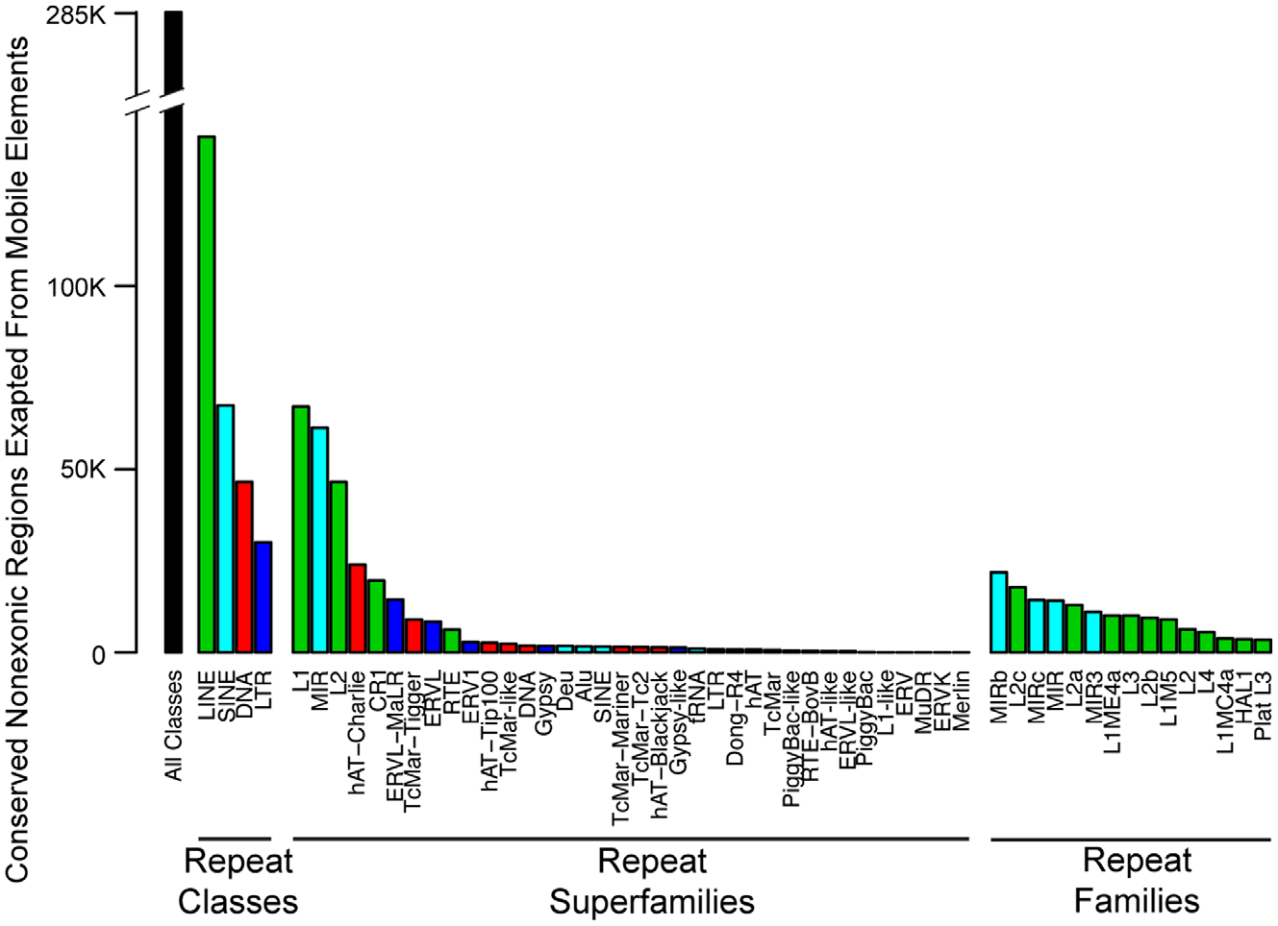
Contribution of mobile element classes, superfamilies, and families. We plotted the number of CNEEs exapted from each repeat class and superfamily, as well as the top contributing families. The superfamilies and families are colored to match the class they belong to. LINE insertions are the class that is creating the most putative regulatory elements. This class is largely composed of the L1 and L2 superfamilies, which have both made large contributions. There is not much statistical power to identify recently inserted sequence as conserved. For this reason, the amount of functional sequence contributed by mobile element superfamilies with recently active members will be an underestimate.

**Figure 7:**
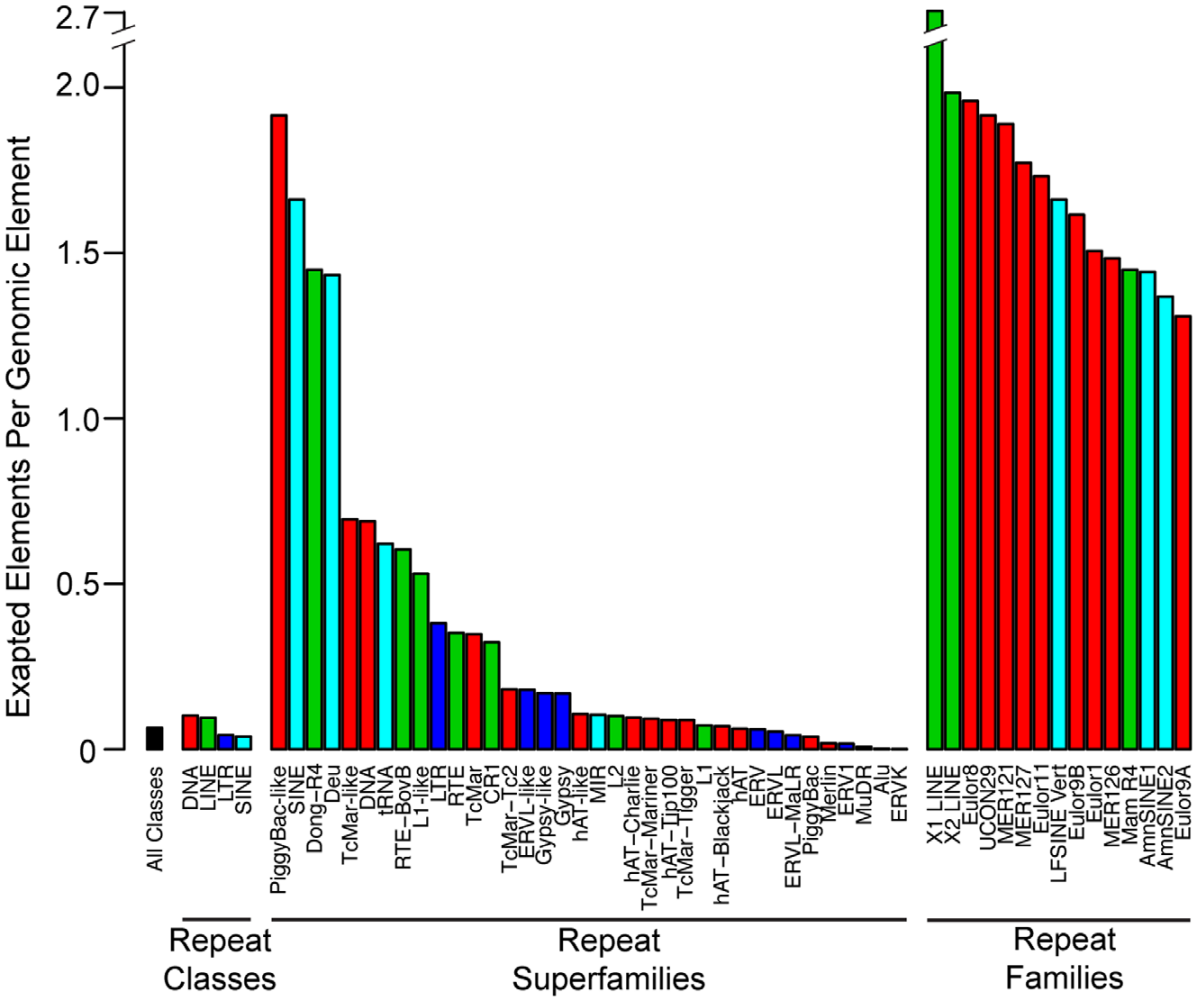
Contribution of mobile element classes, superfamilies, and families relative to their abundance. We plotted the number of exapted instances per genomic instance for classes and superfamilies, as well as the top ranked mobile element families. We colored each superfamily and family to represent the class to which it belongs. Mobile element superfamilies with recently active members will have their contribution underestimated. This is due to the limited statistical power to detect regions evolving under purifying selection when only a few closely related orthologs are available.

Some mobile element superfamilies have provided more putative regulatory sequence than others. The L1 superfamily of LINEs appears to have contributed the largest number of CNEEs to the human genome (Figure 6 and Table 1). This may be expected since L1s have almost 1 million copies in the human genome and account for more than 1 out of every 6 bases. The mobile elements that contributed the greatest number of CNEEs, relative to their copy number in the genome, are all ancient superfamilies that have not been recently active on the human lineage. The top four superfamilies in terms of relative CNEE contribution (Figure 7) are also the top four superfamilies in terms of percentage of their insertions predating the ancestor of placental mammals (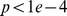, hypergeometric test). For ancient superfamilies, the insertions not under selection have disappeared due to neutral decay, leaving only the slowly evolving exapted copies (Figure 7 and Table 1). It is often difficult to infer the consensus sequence of a mobile element from only a handful of ancient exapted copies. This leads to these ancient exaptations either being putatively placed in a family or having their annotation come from another species where the repeat is still active [21], [59]. The latter was the case with the DeuSINE, which was found to have a near-ancestral version still active in the coelacanth [60]. The DeuSINE was active so long ago on the human lineage that there are more CNEEs attributed to their insertions than there are insertions. Often seeing multiple conserved elements within a single DeuSINE insertion exemplifies that with the 29 mammalian genomes we now have sufficient resolution to not only see that an insertion is evolving under purifying selection, but we can also interrogate exactly which sections of the insertion are under constraint. In the case of the DeuSINE, we see that when an exaptation event happened, it often placed more than one section of the consensus under selection.

We have limited statistical power to detect very recent exaptation events. As a mobile element insertion happens closer to the present day, we have less orthologous sequences in other species and therefore less branch length to notice a resistance to mutations. Many of the recently active mobile elements may be depositing functional sequence, but we will be unable to detect these exaptations. For this reason, many of the mobile elements with few exaptations per genomic instance are recently active (Figure 7).

### Mobile Elements Carry Functional and Nearly-functional Regulatory Elements

With mobile element insertions contributing at least 47% of the extant human genome, we would expect a number of CNEEs would arise out of mobile element insertions by chance, just as can happen with neutrally evolving DNA. If this is the only process by which mobile elements create functional sequence for the host genome, then we would expect the probability of a base position in the consensus coming under selection to be directly proportional to how often that base appears in the genome. However, if a mobile element insertion harbors elements that are functional in the host, nearly-functional, or in some way preferential to the molecular machinery of the host that interacts with DNA, we would expect these bases in the consensus to be overrepresented in the exapted copies relative to the genomic background. In a previous study, we showed that for many mobile elements there is a bias as to where exaptation events happen along the consensus sequence, a finding consistent with the host co-opting functional, pre-functional, or preferential sequences carried by the mobile element [50].

We have detected 259 regions of consensus sequences that are more than twice as likely to be exapted than would be expected from their genomic prevalence. Each peak is based on data from at least 40 exaptation events to avoid small sample sizes. These 259 sections of consensus sequences have an average length of 11 base pairs and delineate regions in the consensus sequence that are more likely to be utilized by the host genome after insertion. To better understand the significance of evolutionary constraint repeatedly occurring in the same region of the mobile element consensus, we randomly placed the set of CNEEs throughout the genome 1000 times. During these 1000 trials only 147 peaks of 2X overrepresentation occurred by random chance, i.e. an average of 0.15 overrepresented peaks per genome. This contrasts with the 259 peaks of 2X overrepresentation we detect in the extant human genome.

It is possible that these preferentially exapted regions of the repeat consensus contain generally useful characteristics for a section of regulatory DNA, such as high GC content [2], a DNA structure easily accessible for protein binding [61], or a general predisposition to be methylated [62]. The alternate explanation is that the mobile element contributes a specific binding site which is then used by the host [29], [30]. In the case of the former, the human paralogs representing the peak will have diverged under different selective constraints and therefore share few similarities in the extant human genome. In the case of the latter, the human paralogs will have been evolving under a similar selective constraint, much as orthologs after speciation.

**Figure 8:**
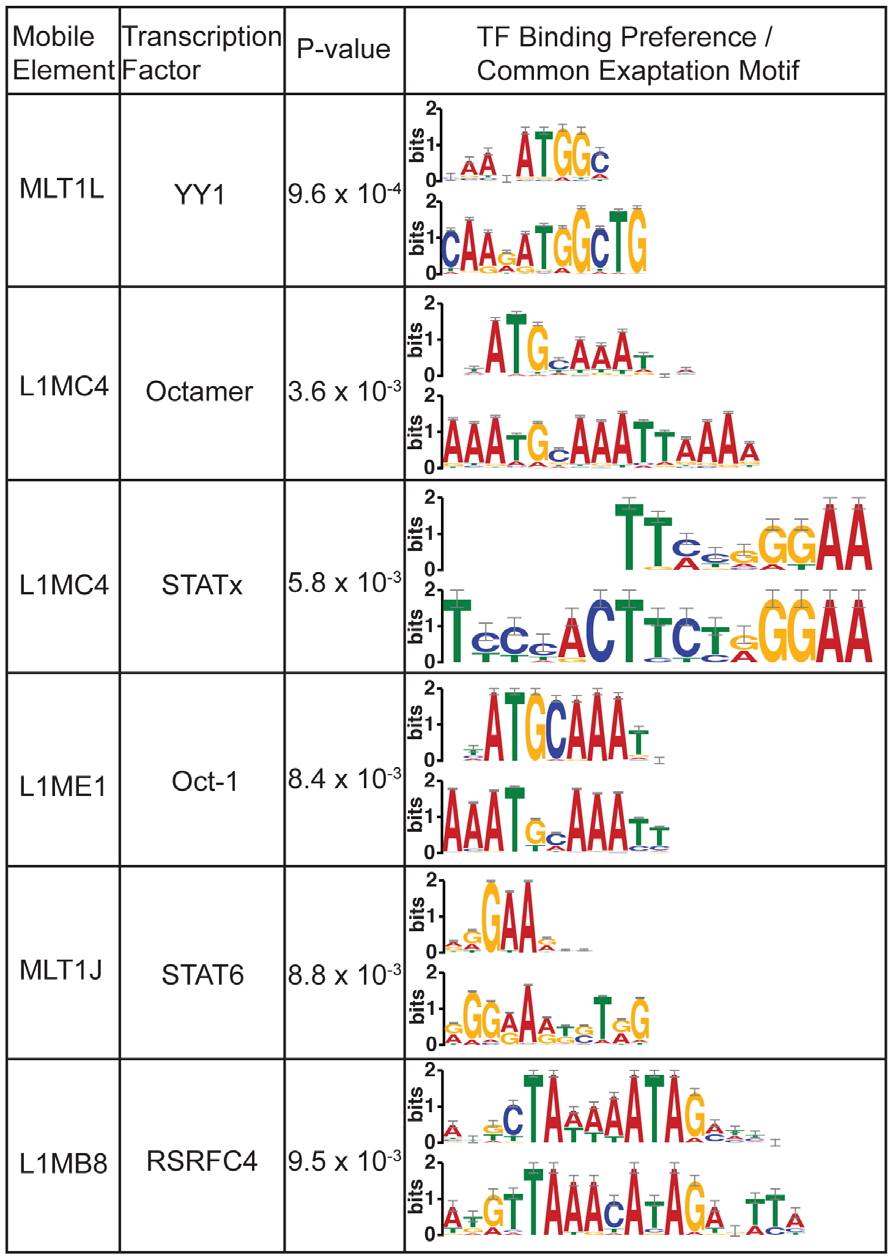
Paralogous instances of mobile elements show selective pressures matching transcription factor binding preferences. We hypothesized that when a particular region of the mobile element is repeatedly exapted, it may be used to perform the same function in paralogous instances. We collected sequences in the human genome representing families of paralogs, that all originated from the same bases of a mobile element insertion. We used MEME [63] to define a motif for this family that represents the selective pressure acting on these insertions after their exaptation by the host. In 6 cases this motif has a significant match to the binding preference of transcription factors (p-values are corrected for multiple tests). These results are consistent with mobile element consensus sequences spreading functional, or near-functional, transcription factor binding sites throughout the genome, which are then exapted by the host. A more detailed analysis of one of these matches is shown in Figure 9.

Just as the orthologs of a binding site conserved across species may be aligned to elucidate the preference for A, C, G, and T at various positions, the same can be done with paralogous exaptations. For each section of the consensus where exaptations preferentially occur, we used MEME [63] on the human paralogs to define a motif common to most, or all, of the exaptations. 225 of the 259 peaks are defined by a motif greater than 8 base pairs in length and an e-value less than 0.01, after correction for multiple tests (see Methods). We then compared the sequence motifs from the human paralogs against known vertebrate transcription factor binding profiles (see Methods). There are 6 matches between motifs defined by paralogous exaptations in the human genome and known binding motifs for transcription factors (Figure 8). All 6 of these matches between paralogous motifs and TF binding motifs have a corrected p-value less than 0.01.

**Figure 9:**
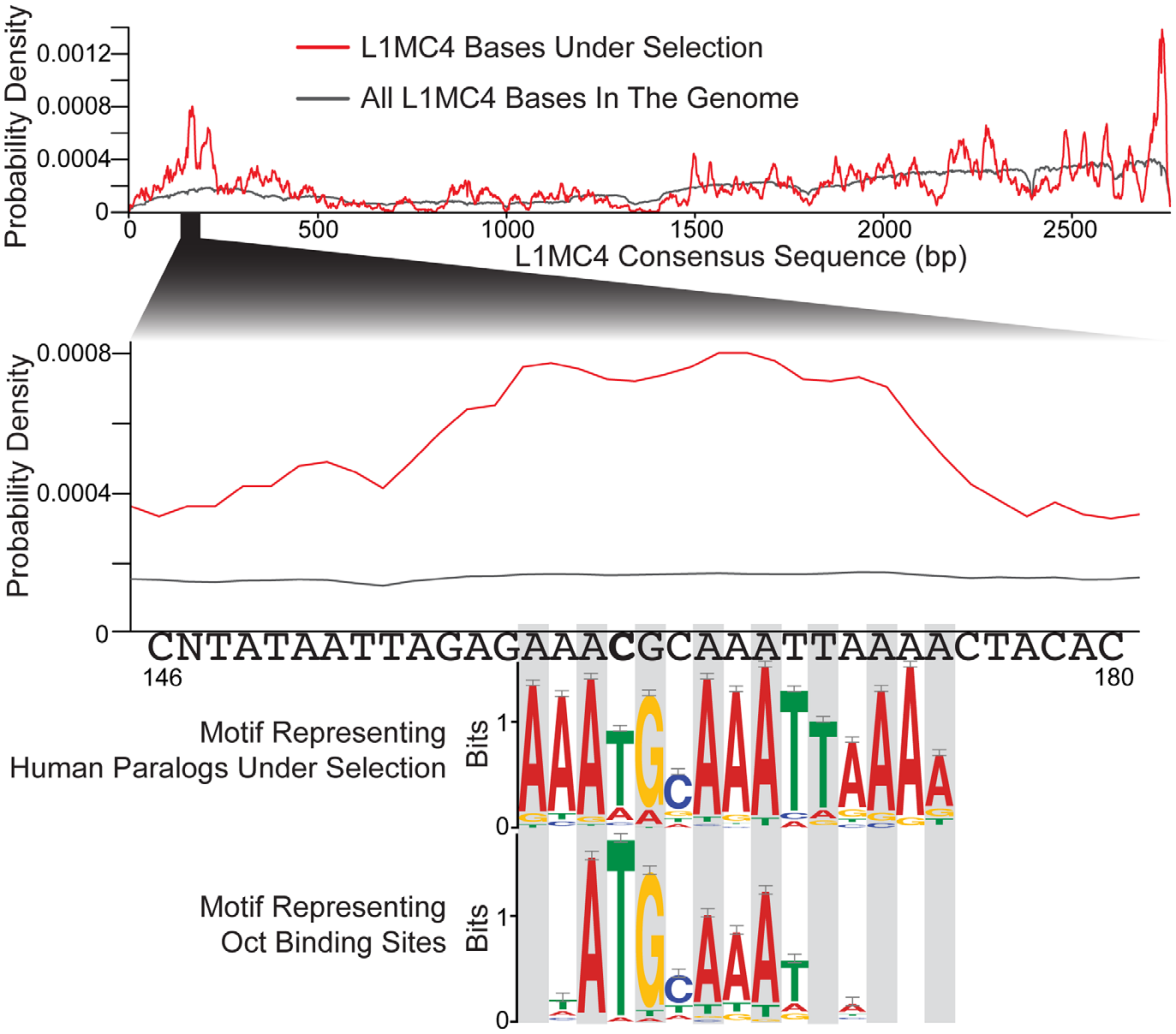
L1MC4 may be a fecund source of octamer binding sites. The probability density of each base in the L1MC4 consensus being present in a genomic copy (gray) or an exapted copy (red) is plotted (top plot). When zooming in to the second highest peak of exaptation probability we show the consensus sequence as well. By using motif finding software on the exaptation events in the extant human genome that contributed to this peak, we obtained a profile describing the selection acting on paralogous exaptations of this small region. This profile is easily alignable to the consensus, but it is interesting to note the ‘C’ in the consensus (bold type) that routinely changes to a ‘T’ in the exaptations. The profile describing the selective pressure acting on these paralogs is similar to the octamer binding profile, which is consistent with this section of the L1MC4 consensus often being exapted on the human lineage to act as a binding site for a member of the octamer family of proteins.

An example of human paralogs defining a motif that matches a known transcription factor binding profile is the L1MC4 element, which appears to have a section of its 5′ end conserved to act as a binding site for one of the octamer transcription factors (Figure 9). The consensus of the L1MC4 element does not contain an octamer binding site that is then retained after insertion, but rather it contains a nearly-functional site that is a single substitution from being functional. The substitution is a CpG dinucleotide undergoing a transition to a TpG dinucleotide, which is a a common substitution that happens at 12 times the normal rate of transitions [64]. While consensus L1MC4 instances do not match the octamer binding profile upon insertion, it seems that they are poised to bind an octamer family transcription factor after a single commonly-occurring mutation that may then be driven to fixation by selection. A similar phenomenon has been shown in Alu elements, where deamination may result in p53 binding sites [65].

### Estimating the Contribution of Mobile Elements to Gene Regulatory Innovations

We have conservatively estimated a lower bound of 11% on the fraction of CNEEs deriving from mobile element insertions. A more accurate estimate is obtained by calculating the CNEEs appearing on a single branch and determining how many of these CNEEs have their origins in mobile element insertions. We have chosen the branch of the human lineage following the split with marsupials (opossum) and prior to the speciation of atlantogenata (elephant). We selected this branch because it is close enough to the present that we understand many of the mobile elements that were active at the time, but ancient enough that we can easily detect selection based on orthologous regions in other species. On this branch we calculate that ∼19.2%, almost 1 in 5, of the CNEEs are the product of an exaptation event involving a mobile element. This is an increase from the ∼16% that was estimated for the same branch at the time when the opossum genome was first published [34]. To test the robustness of this estimate to the method of repeat annotation we repeated the calculation using the Censor [66] software package. This yielded an even higher, yet similar, estimate of ∼19.6%. While this appears to be a robust estimate for the ∼40My of the branch, it is unclear how generalizable the contribution of transposons over this time interval is to all of human evolution. It is possible that the influx of mobile elements, regulatory potential of mobile elements, and rate of regulatory innovations has not been consistent through time. Large changes in these variables may lead to an non-uniform contribution of mobile elements to regulatory innovations during human evolution.

## Discussion

The availability of 29 mammalian genomes has enabled us to explore the evolutionary mechanism of host genomes exapting fragments of mobile elements to act as putative gene regulatory sequence at a more detailed level. Compared to earlier studies that had access to only a handful of mammalian genomes [34], [50], we can now detect more than 6 times as many exapted bases (∼7 Mb), more than 25 times as many exapted elements (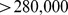), and the estimate of CNEEs being created by this mechanism has risen to nearly 20 percent. This estimate should continue to increase as the research community annotates additional mobile elements. Perhaps most surprising is that more than 6% of mobile element insertions present in the reference assembly appear to harbor sequence that is under selection for a non-exonic function. With many mobile elements insertions being too young to detect cross-species constraint, this percentage should only increase as additional methods are used to identify functional sequence.

In 1971 Britten and Davidson hypothesized that the large amount of repeated DNA in animal genomes, which we now know to be largely from mobile element insertions, may contain the regulatory information needed to express genes in concert during development [67]. This suggests that some paralogous human instances of mobile element insertions will have the same function, and therefore be under similar selective constraint. One example of this is the dispersion of the MER20 element in placental mammals helping to create whole new cell types during development [32]. We provide new evidence supporting this view by computationally identifying common selective constraints in some mobile element insertions that match known binding profiles of transcription factors. This method may serve as a useful means to direct experiments investigating how a transcription factor’s regulatory network may be built or modified by the exaptation of mobile element fragments [29]–[31].

While mobile elements will likely never recover their original name of “controlling elements,” we believe that recent work by many researchers is helping to show that McClintock’s original term was applicable, and perhaps only suffered because her insights were ahead of her time.

## Methods

### Alignment

**Figure 10:**
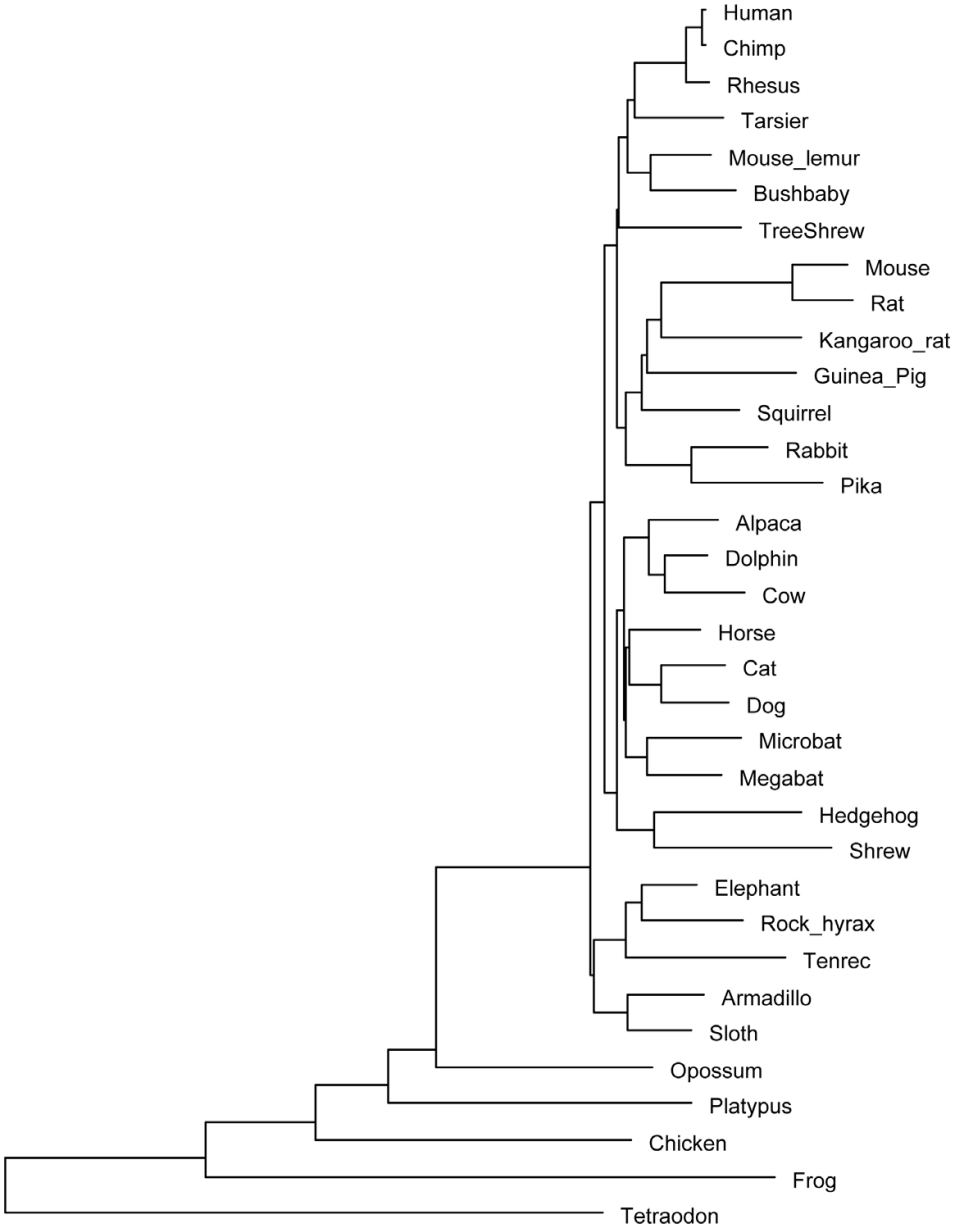
Phylogenetic tree of 29 placental mammals including some outgroup species. We used the topology from the 2x Mammals Consortium [1]. We have included opossum, platypus, chicken, frog, and tetraodon as outgroup species.

We used the alignment and species tree (Figure 10) computed by the 2x Mammals Consortium [1]. The alignment was referenced on the human genome and contains the following 29 placental mammals: human (hg18), chimp (panTro2), rhesus (rheMac2), tarsier (tarSyr1), mouse lemur (micMur1), bushbaby (otoGar1), tree shrew (tupBel1), mouse (mm9), rat (rn4), kangaroo rat (dipOrd1), guinea pig (cavPor3), squirrel (speTri1), rabbit (oryCun1), pika (ochPri2), alpaca (vicPac1), dolphin (turTru1), cow (bosTau4), horse (equCab2), cat (felCat3), dog (canFam2), microbat (myoLuc1), megabat (pteVam1), hedgehog (eriEur1), shrew (sorAra1), elephant (loxAfr2), rock hyrax (proCap1), tenrec (echTel1), armadillo (dasNov2), and sloth (choHof1).

### Defining a Set of CNEEs

Our set of CNEEs is the subset of conserved elements that have no overlap with bases appearing in mature transcripts. The set of conserved elements was defined by the 2x Mammals Consortium [1] using phastCons [16] to analyze a multi-species alignment of 29 mammals. We removed all the elements in this set that had any overlap with tracks in the UCSC genome browser [68] that depict mature transcripts: UCSC Genes [69], CCDS [70], RefSeq Genes [71] from humans and other species, MGC Genes [72], TransMap [73], Vega Genes and Pseudogenes [74], Ensembl protein-coding genes and non-coding genes [75], Exoniphy [76], RNA Genes, Yale Pseudogenes [77], UCSC Retrogenes and Pseudogenes [78], and sno/miRNA [79].

### Derived Allele Frequency Spectra

We based our derived allele frequency analysis on the July 2010 data release of the 1000 Genomes Project [80]. We used only segregating single nucleotide polymorphisms in the Yoruban population where the 1000 Genomes Consortium had provided an annotation of the ancestral allele. To define a reliable set of protein-coding regions, we used the intersection of protein-coding exons annotated by UCSC genes [69], CCDS [70], RefSeq genes [71], and Ensembl genes [75]. To define our set of intronic regions we used the intersection of introns from the same gene annotation projects. To determine if spectra were significantly shifted relative to each other we used the Mann-Whitney U test.

### Defining a Set of Bases with Mobile Element Ancestry

To identify bases in the extant human genome that have origins in a mobile element insertion we used both RepeatMasker v3.2.7 (http://www.repeatmasker.org/) and Censor v14.01 [66]. Both programs were run with sensitive parameter settings (RepeatMasker, -s; Censor, -mode sens) on the March 2006 assembly of the human genome (hg18) and using repeat libraries from RepBase [81]. We extracted the annotations for SINEs, LINEs, DNA transposons, and LTRs to use in our analysis. A CNEE was annotated as having mobile element ancestry if the majority of its bases were annotated as a mobile element insertion. The results are not largely dependent on this threshold since more than 90% of the CNEEs with a majority of their bases annotated as a mobile element are entirely annotated as a mobile element.

### Calculating the Rate of Evolution for Each CNEE

We represented the rate of neutral evolution as a time reversible matrix defining the probability of all base substitutions and a tree whose branch lengths define the average substitutions per site based on four-fold (4d) degenerate sites in codons. We used one tree to represent the neutral rate on chromosome X and another representing 4d sites in the rest of the genome [1]. To calculate the rate of evolution for each CNEE we extracted the CNEE and its orthologs from a multi-species alignment of 29 mammals [1]. We then used phyloFit [82] to scale a neutral tree by a single constant to fit the alignment of the CNEE and its orthologs. The scale constant is equal to the fraction of the neutral rate that describes the CNEE’s rate of evolution.

### Dating Exaptation Events

To date exaptation events, we used a multi-species alignment of 29 mammals [1], as well as opossum, platypus, green lizard, chicken, tetraodon, stickleback, and fugu. For each exaptation we begin with the most divergent group of species and calculated if half, or more, of the bases in the CNEE were aligning to any species in the group. This was iterated with progressively closer species until more than half of the CNEE bases were present in the ancestor of human and the group of species being investigated. The exaptation event was placed on the branch of the human lineage above the ancestor that appears to have contained at least half of the CNEE bases.

### Aligning Exaptation Motifs to those of Transcription Factors

We used MEME [63] on paralogous instances of highly-exapted mobile element regions to define a motif common to most, or all, of the exaptations. We then used Tomtom [83] to compare these motifs representing the constraint experienced by these commonly exapted regions to known vertebrate transcription factor binding profiles from Transfac [84].
